# Supplementary material for: Adhesion energy controls lipid binding-mediated endocytosis
Source: Nat Commun. 2024 Mar 29;15:2767. doi: 10.1038/s41467-024-47109-7 (PMC10980822; doi:10.1038/s41467-024-47109-7)
Supplement: Supplementary file 1 — Supplementary Information [file 41467_2024_47109_MOESM1_ESM.pdf]

Adhesion energy controls lipid binding-mediated endocytosis

Raluca Groza[1], Kita Valerie Schmidt[1,2], Paul Markus Müller[1], Paolo Ronchi[3], Claire Schlack-Leigers[1], Ursula Neu[1], Dmytro Puchkov[4], Rumiana Dimova[2], Claudia Matthaeus[5,6], Justin Taraska[5], Thomas R. Weikl[2] and Helge Ewers[1*]

[1] Institute of Biochemistry, Freie Universität Berlin, Thielallee 63, 14195, Berlin, Germany
[2] Max Planck Institute of Colloids and Interfaces, Potsdam Science Park, Am Mühlenberg 1, 14476 Potsdam, Germany
[3] Electron Microscopy Core Facility, European Molecular Biology Laboratory, 69117 Heidelberg, Germany
[4] Leibniz-Forschungsinstitut für Molekulare Pharmakologie (FMP), 13125 Berlin, Germany
[5] National Heart Lung and Blood Institute, National Institutes of Health, Bethesda, Maryland 20892, USA
[6] present address: Institute for Nutritional Science, University of Potsdam, Arthur-Scheunert-Allee 114-116, 14558 Nuthetal, Germany
*corresponding author

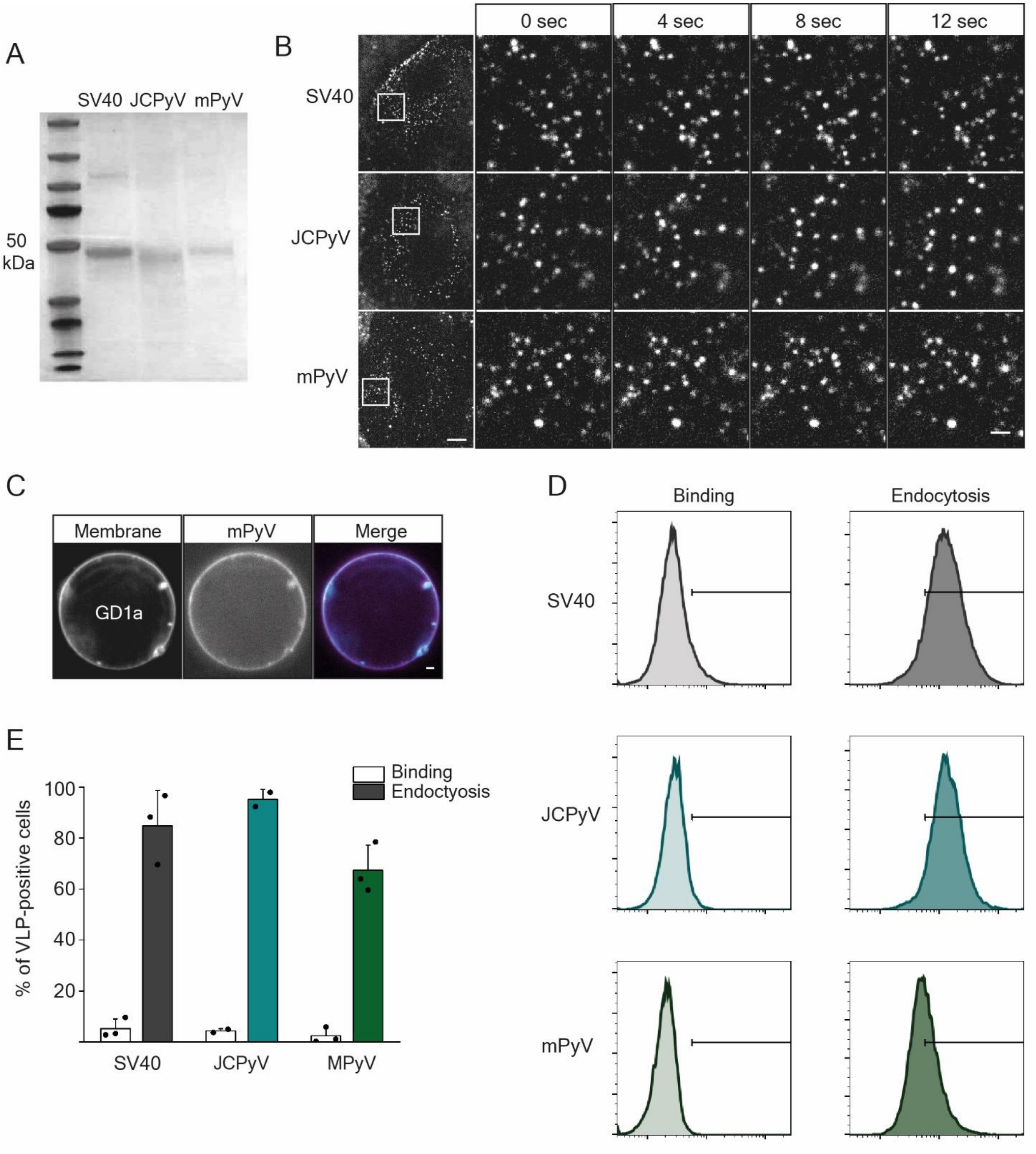

Figure S1. Characterization of polyomaviridae virus-like particles. A) SDS-Page gel showing the single capsid protein composition of the polyoma VLPs. **B)** Fluorescence micrographs of polyoma VLP binding to the membrane of CV1 cells for 30 min at at 4 ˚C before imaging live on a spinning disk confocal microscope at 37 ˚C. Left panels: overview of the cells with bound VLPs as indicated. Scale bar is 10 µm. Right panels: time-course fluorescence micrograph insets of the regions of interest indicated in the left panels for the respective polyoma VLPs diffusing on the membrane. Scale bar is 2 µm. **C)** Spinning disc confocal fluorescence micrographs of MPyV bound to GUVs containing GD1a receptor ganglioside. 2 µg of VLP was incubated for 1 h at RT with GUVs (98% DOPC, 1% GD1a, 1% β-BODIPY FL C12-HPC dye) and imaged at the equatorial plane. Scale bar is 2 µm. **D)** Histograms of the binding or endocytosis of the marked VLPs in CV1 cells, as determined from flow cytometry measurements. Left panel: binding assay, cells were incubated with the indicated VLPs at 4 ˚C for 45 min before acid wash to remove the cell surface-bound VLP fraction. Right panel: endocytosis assay, cells were incubated with the indicated VLPs at 37 ˚C for 1 h before acid wash. Measured is the fluorescene intensity of individual cells, for at least 5000 cells/sample, n = 2 independent experiments. **E)** Quantification of polyoma VLP binding and endocytosis from the flow cytometry measurements represented in D), means ± S.D., from at least 5000 cells/sample from n = 2 independent experiments.

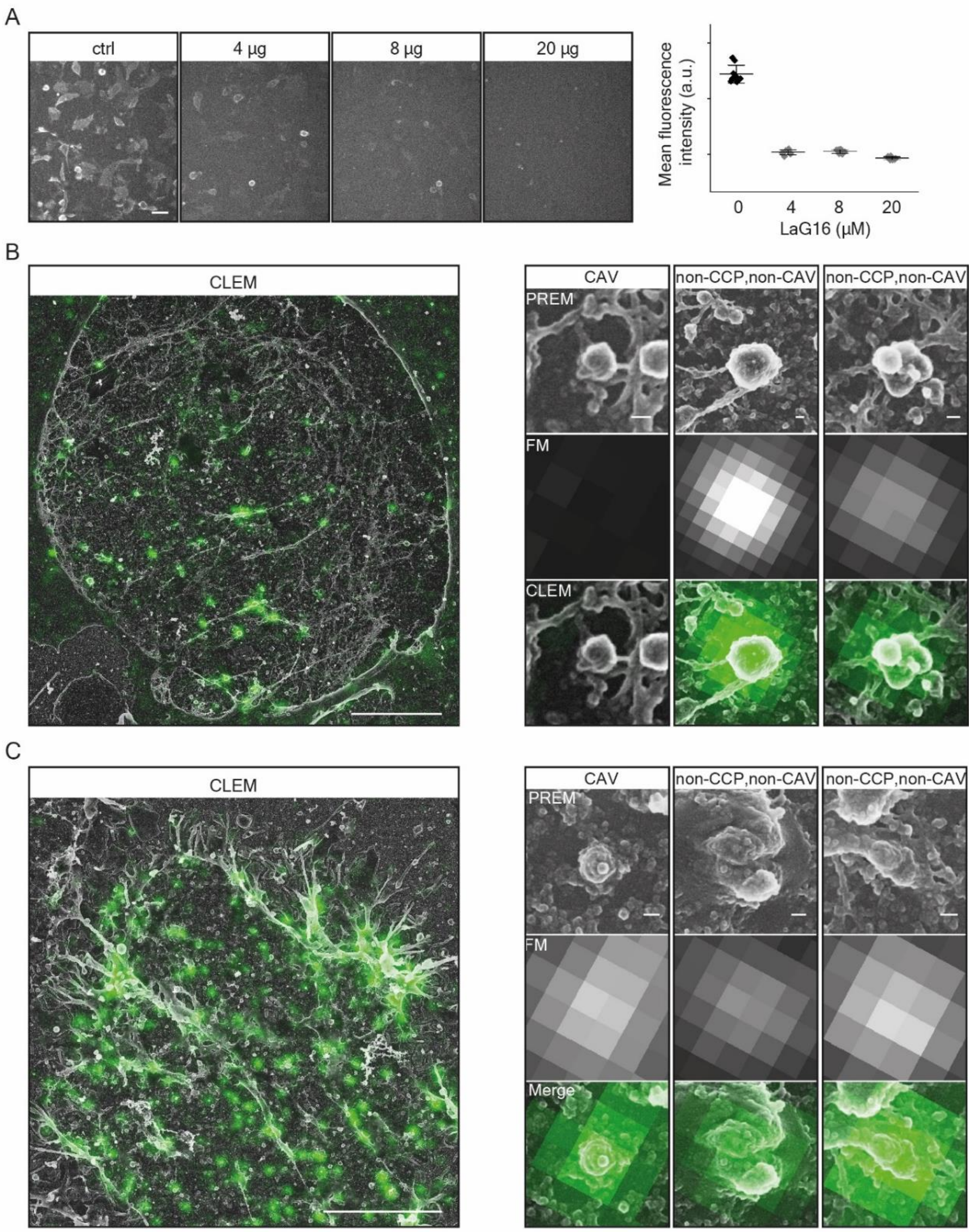

Figure S2. Early surface interaction of GFP GEMs. A) Left panel: Fluorescence micrographs of GEMs bound to the surface of the cells after pre-mixing with the indicated concentrations of recombinant LaG16 nanobody. Right panel: Quantification of the fluorescence intensity of the micrographs represented in the left panel. Fluorescence intensity means ± S.D. per Z stack from 38 cells from n=1 independent measurement. Scale bar is 50 µm. **B) and C)** (left panel) Overview of correlative fluorescence, platinum-replica electron microscopy micrographs of plasma membrane sheets generated after unroofing of cells incubated with GEMs (green). Scale bars are 5 µm. (right panel) Shown are typical, GEM-negative (top) or a GEM-positive (bottom) caveola and clathrin- and caveolin-negative endocytic structures containing GEMs (positive for EGFP fluorescence signal). Electron microscopy micrographs are on top, corresponding fluorescence microscopy images of the same field of view are in the middle and the correlative EM images are at the bottom. Scale bars are 50 nm.

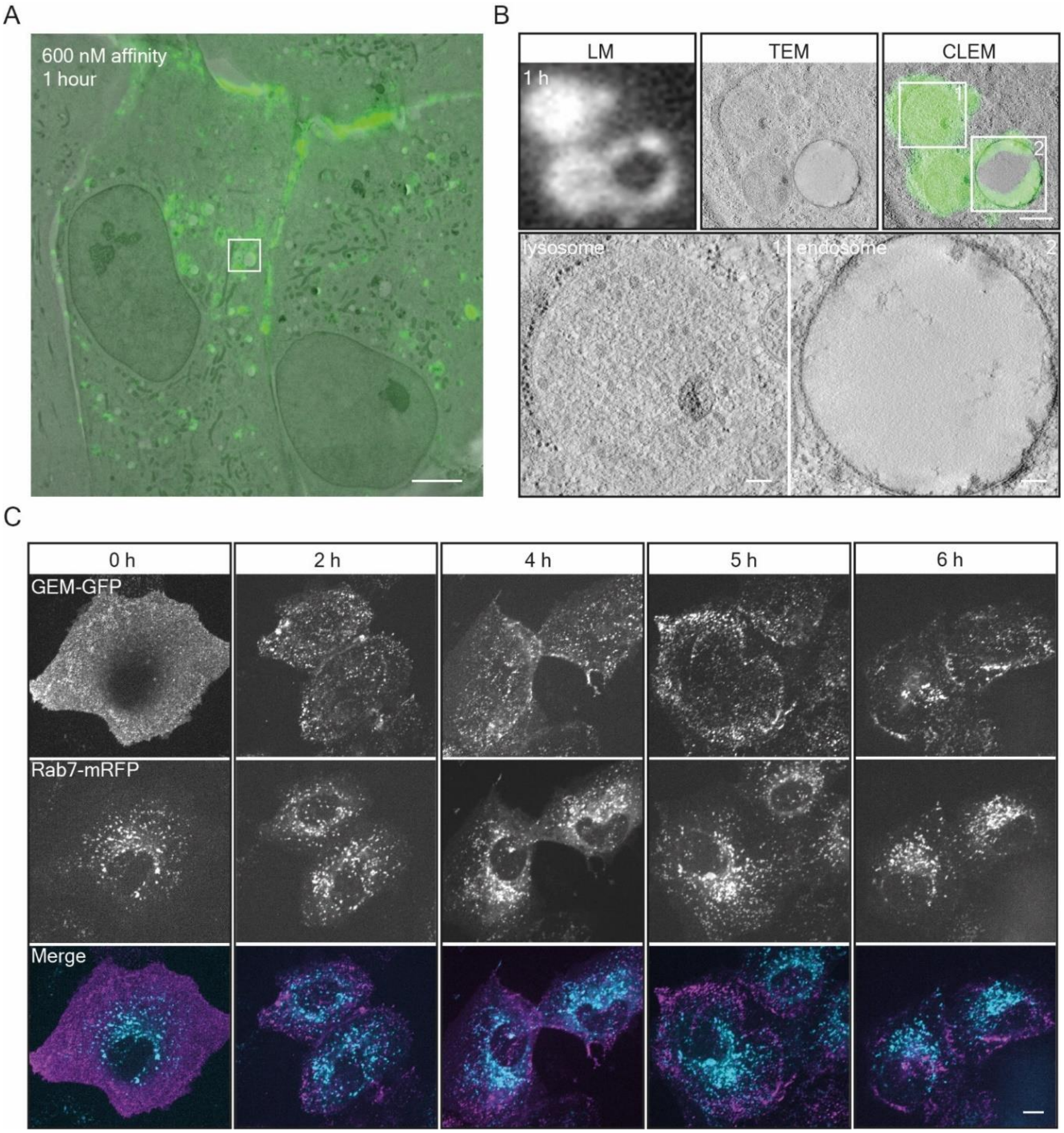

Figure S3. Intracellular trafficking of GFP GEMs. A) Low magnification correlative fluorescence light microscopy and transmission electron microscopy of GEMs internalized in CV-1 cells expressing the 600 nM binding affinity GPI-anchored nanobody. Timepoint 1h after binding. Scale bar is 5 µm. **B)** High magnification correlative fluorescence light microscopy and transmission electron microscopy of GEMs internalized in CV-1 cells expressing the 600 nM binding affinity GPI-anchored nanobody. Top panel from left to right: Fluorescence micrograph of GEMs; transmission electron micrograph of same region; correlative images. Bottom panel: Transmission electron micrograph of inset above. Scale bars are 500 nm for overview and 100 nm for insets. **C)** Fluorescence micrographs from a time-course experiments of endocytosis showing the distribution of GEMs in CV1 cells expressing anti-GFP nanobody and Rab7-mRFP. Cells were incubated with 2 µg of GEMs for the indicated time points at 37 ˚C before live imaging on a spinning disk confocal microscope. Scale bar is 10 µm.

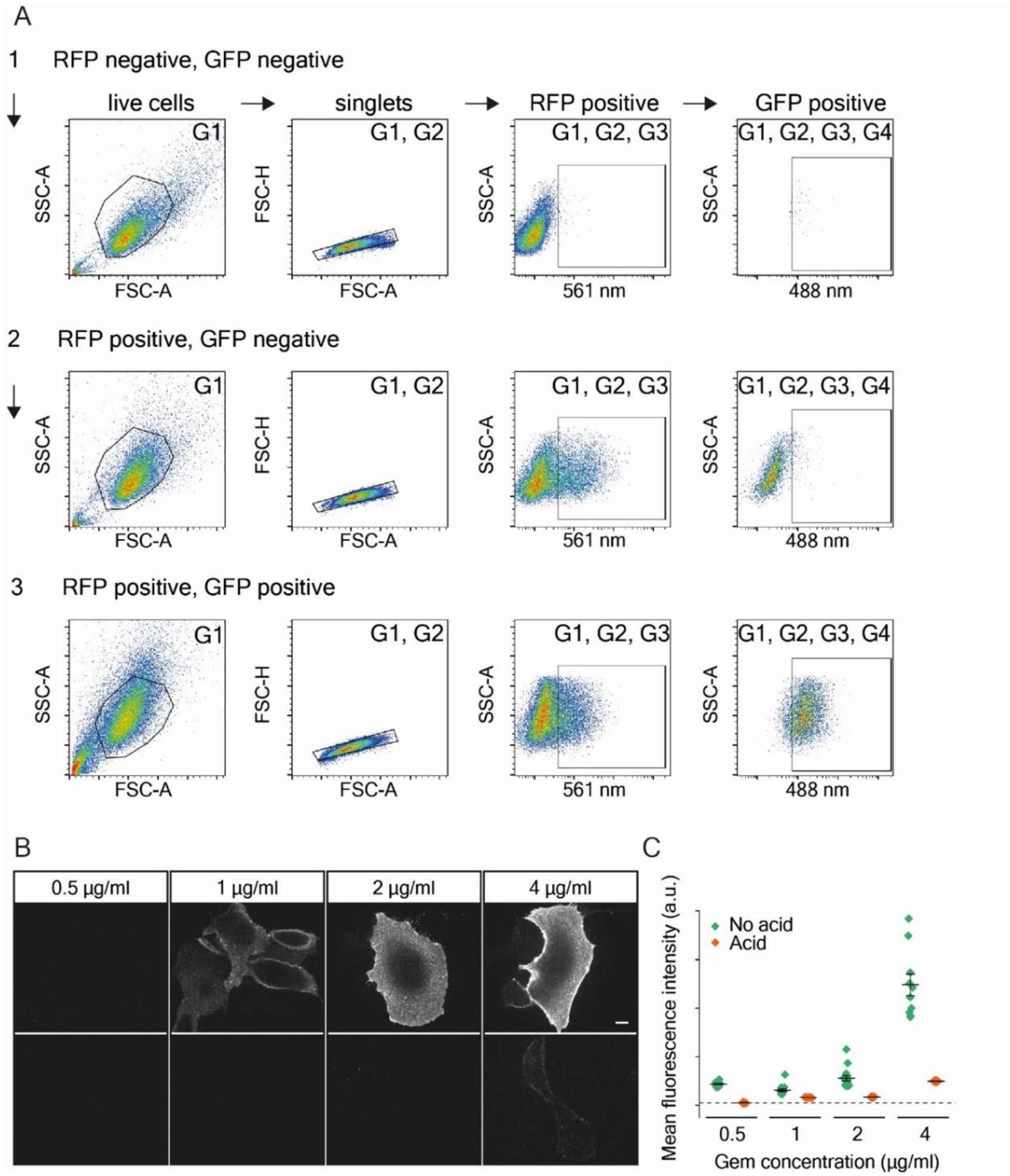

Figure S4. Gating strategy of flow cytometry measurements and validation of internalization. A) Gating strategy for the flow cytometry experiments. In brief, live cell population was always selected first, indicated as G1 (left panels). From the selected live cell population, doublets and aggregates were always removed, indicated as G1, G2 (second panels from the left). Next, the RFP positive population gate was selected according to the RFP negative control in the top panels. This gate is marked as G1, G2, G3 (second panels from the right). Next, the GFP positive population gate was selected according to the RFP positive, GFP negative control shown in the middle panels. This gate is marked as G1, G2, G3, G4 (right panels). A representative sample of GEMs in GPI-anchored anti-GFP nanobody expressing cells is shown in the lower panel, the respective gates as indicated. Figure S6 displays the G1,G2,G3,G4 gate (GEM-GFP positive) as histograms and the corresponding G1,G2,G3 gate (RFP positive) for the RFP co-transfection levels of the same cells. **B)** Fluorescence micrographs of increasing concentrations of GEMs bound for 5 min at 37 ˚C to CV1 cells expressing the 0.036 nM binding affinity GPI-anchored anti-GFP nanobody before (top panel) or after acid wash (lower panel), imaged live on a spinning disk confocal microscope. Scale bar is 10 µm. **C)** Quantification of mean intensity fluorescence of the different concentration of GEMs bound to the cells represented in B) before (green) and after acid wash (orange). The dotted line represents fluorescence background level. Shown are fluorescence intensity means ± s.e.m. from the Z stacks of 108 cells from n=1 independent measurement.

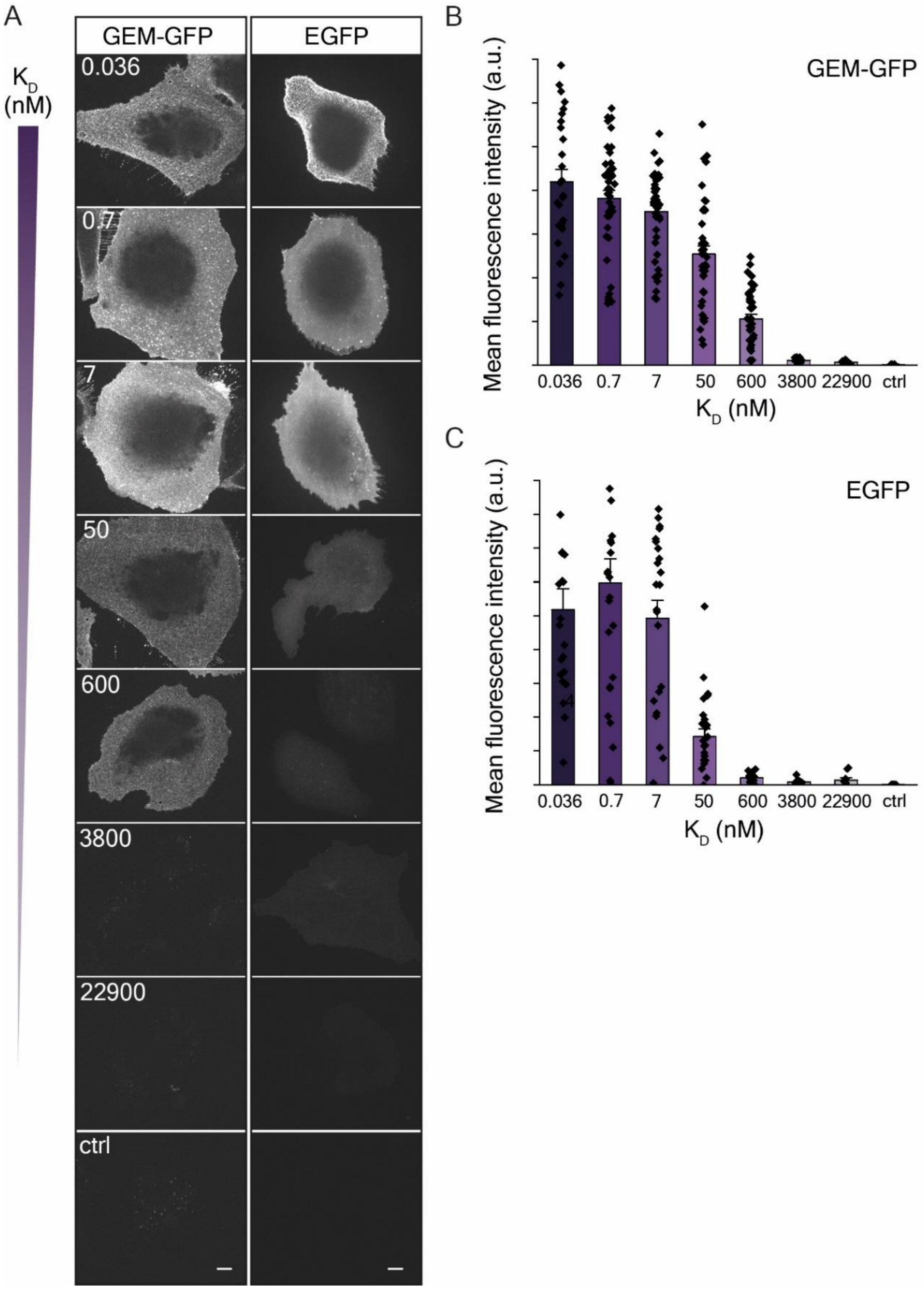

Figure S5. Quantification of cell binding for GFP alone and GFP GEMs. A) Fluorescence micrographs of GEMs (left panel) and recombinant EGFP (right panel) binding to the membranes of CV1 cells expressing the panel of GPI-anchored anti-GFP nanobodies as indicated. Cells were incubated with 2 µg of either GEMs or EGFP for 30 min at 4 ˚C before imaging live on a spinning disk confocal microscope. Scale bars are 10 µm. **B)** Quantification of GEMs binding as a function of receptor affinity determined from the fluorescence micrographs represented in panel A). Shown are fluorescence intensity means ± s.e.m. from the Z stacks of 254 cells from n=1 independent measurement. **C)** Quantification of EGFP binding as a function of receptor affinity determined from the fluorescence micrographs represented in panel A). Shown are fluorescence intensity means ± s.e.m from the Z stacks of 133 cells from n=1 independent measurement.

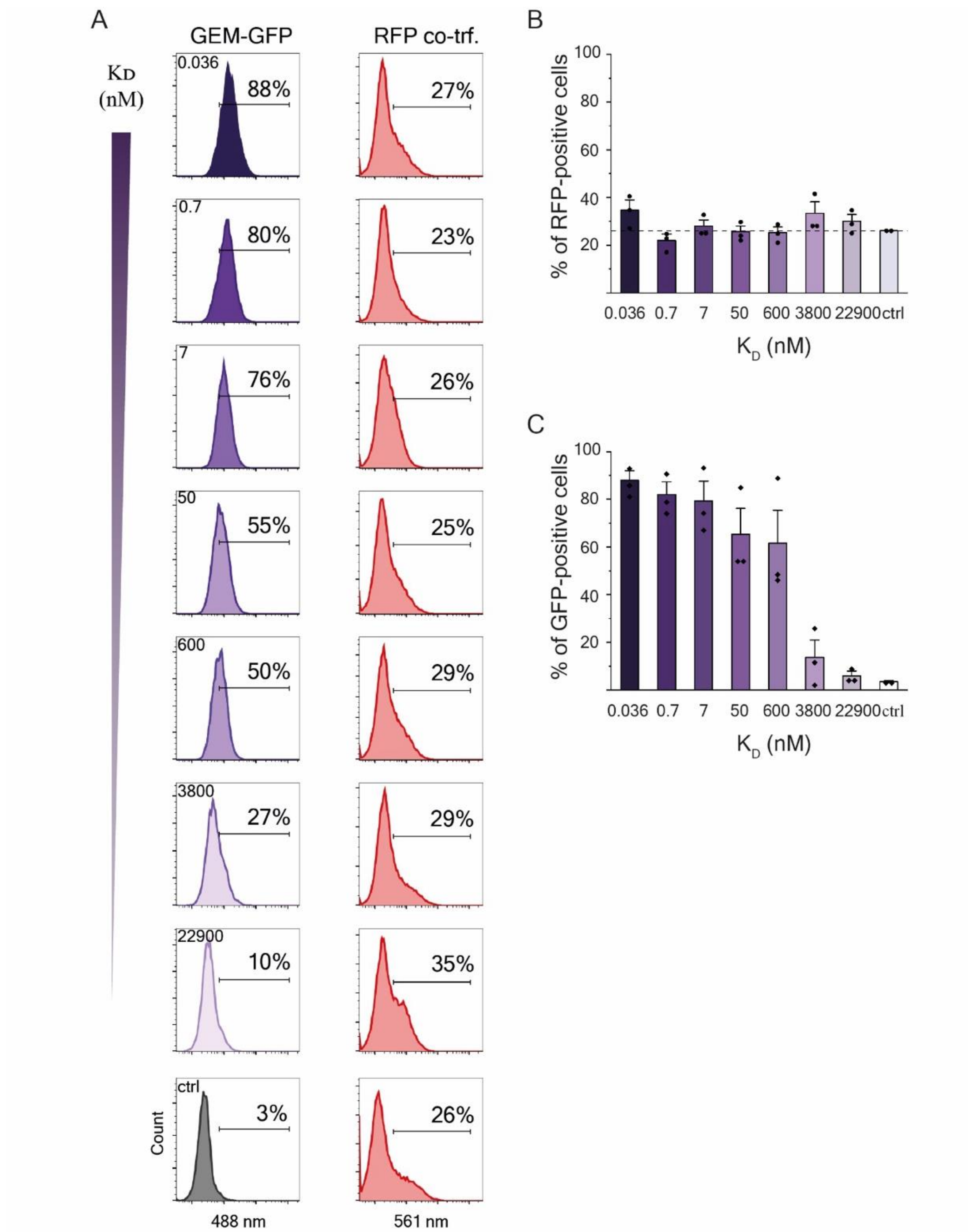

Figure S6. Quantification GFP-GEM positive cells versus RFP positive cells after cotransfection of RFP with GPI-nanobodies for internalization assay. A) Histograms of the GEM endocytosis (left panel) and RFP co-transfection (right panel) in CV1 cells expressing the panel of GPI-anchored anti-GFP nanobodies as indicated, determined from flow cytometry measurements. Cells were incubated with 2 µg of GEM-GFP for 1 h at 37 ˚C before acidic wash to remove all cell surface-bound GEM fraction. The fluorescence intensities of individual cells were then measured for both GEM and RFP channels, for at least 5000 cells/sample. The percentage of either GFP- or RFP-positive cell population from the total amount of cells is marked on the histograms. **B)** Quantification of the percentage of RFP-positive cells from the total amount of cells as a function of receptor affinity, determined by the flow cytometry measurements represented in panel A). Means ± s.e.m. from at least 5000 cells/sample from n = 3 independent experiments. Dotted line represents RFP-positive level of control cells transfected with RFP alone. **C)** Quantification of the percentage of GEM positive cells from the total amount of cells as a function of receptor affinity, determined by the flow cytometry measurements represented in panel A). Means ± s.e.m. from at least 5000 cells/sample from n = 3 independent experiments.

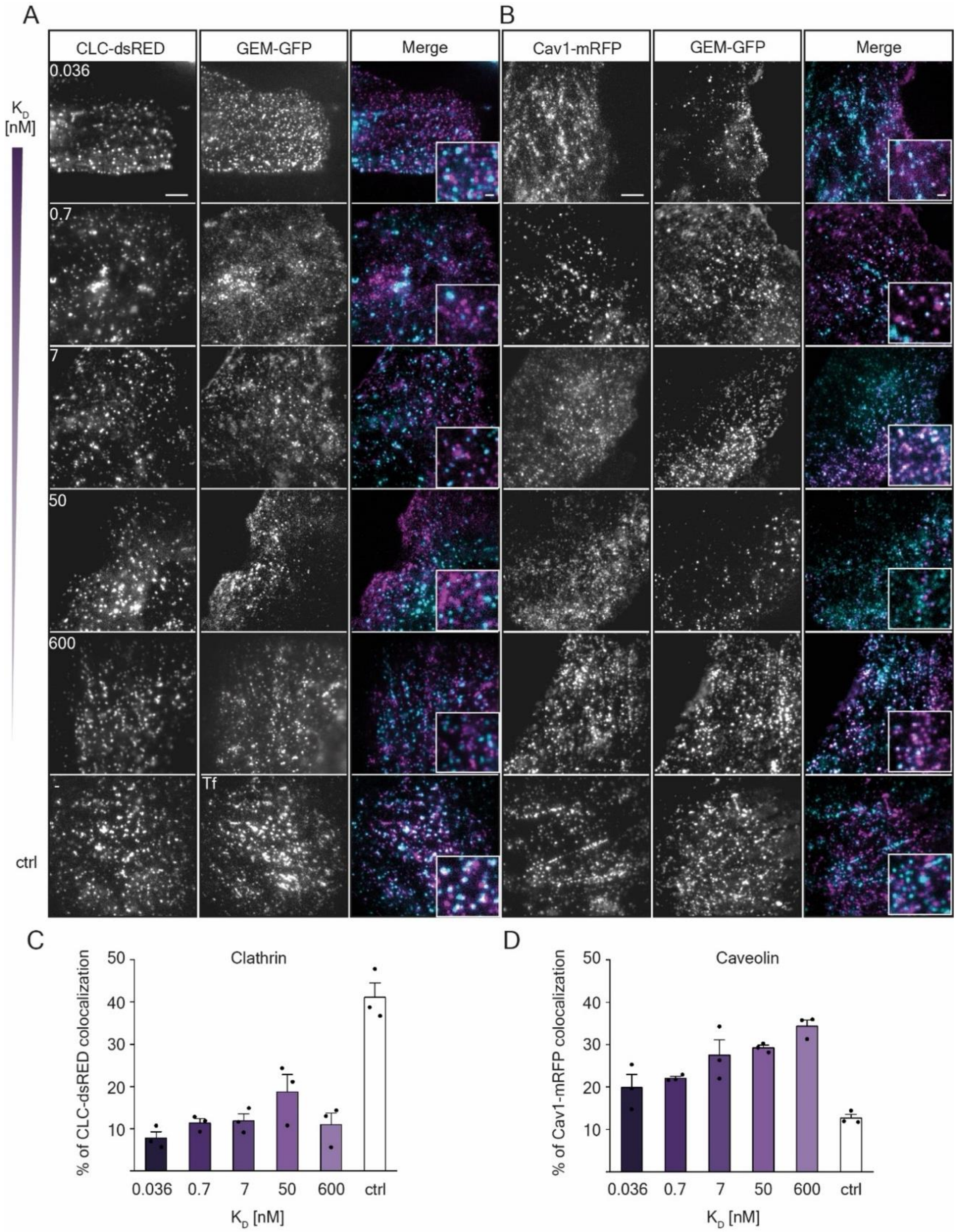

Figure S7. Quantification of GEM-GFP colocalization with clathrin and caveolin in dependence on receptor affinity. A) Fluorescence micrographs of GEMs (magenta) bound to CV1 cells expressing Clathrin-light-chain-dsRED (cyan) and the panel of GPI-anchored anti-GFP nanobodies as indicated. CV1 cells were incubated for 10 min with 2 µg of GEMs at 37 ˚C before time-course live imaging on a TIRF microscope. Images were acquired every 1 min for 6 min in total. Scale bar is 5 µm for overview and 1 µm for inset. **B)** Fluorescence micrographs of GEMs (magenta) bound to CV1 cells expressing Caveolin1-mRFP (cyan) and the panel of GPI-anchored anti-GFP nanobodies as indicated. CV1 cells were incubated for 10 min with 2 µg of GEMs at 37 ˚C before time-course live imaging on a TIRF microscope, as described in A). Scale bar is 5 µm for overview and 1 µm for inset. **C)** Quantification of colocalization between GEMs and CLC-dsRED from 6 timepoints/cell taken at 1 min interval, means ± s.e.m. from at least 9 cells/ sample from n = 3 independent experiments. **D)** Quantification of colocalization between GEMs and Cav1-mRFP from 6 timepoints/cell taken at 1 min interval, means ± s.e.m from at least 9 cells/sample from n = 3 independent experiments.

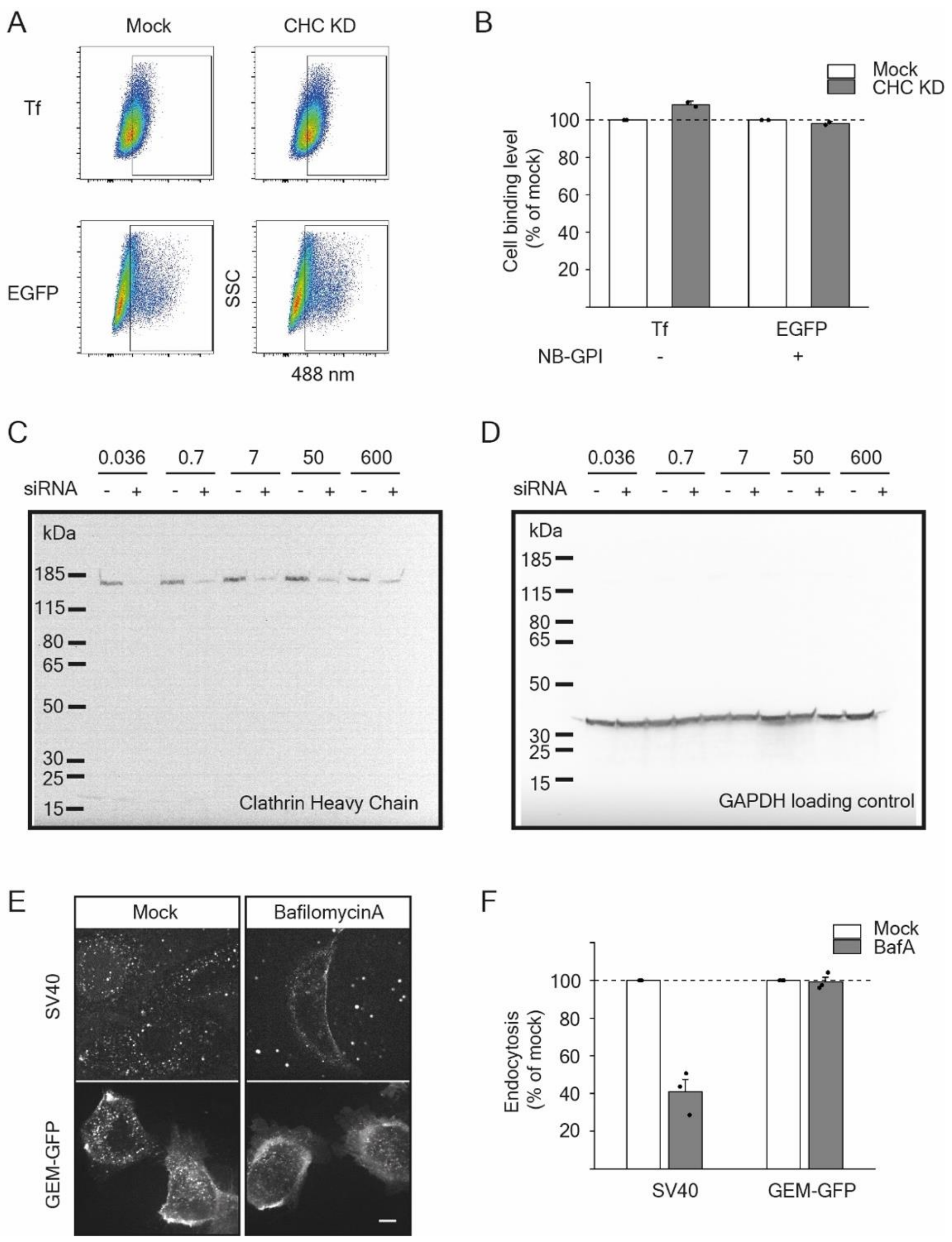

Figure S8. Control experiments for pharmacological inhibitors and siRNA-mediated knockdown. A) Fluorescence intensity dot blots from flow cytometry measurements of Transferrin-AF488 binding (top panel) to non-transfected CV1 cells or of EGFP binding (bottom panel) to CV1 cells expressing the 0.036 nM binding affinity GPI-anchored nanobody receptor. Cells were either mock treated (left panels) or treated with genetic inhibitors (siRNA) against clathrin-heavy-chain (right panels). The cells were incubated with 2 µg of either Transferrin-AF488 or recombinant EGFP for 45 min at 4 ˚C before flow cytometry measurements. **B)** Quantification of Transferrin-AF488 and EGFP binding to cells upon mock or siRNA treatment, as represented in the flow cytometry dot blots in panel A). Measured is mean fluorescence intensity of at least 5000 cells/sample from n = 2 independent experiments, means ± s.e.m. **C)** Scan of uncropped western blot showing CHC levels in cells expressing the panel of GPI-anchored anti-GFP nanobody constructs as indicated. Cells were either mock treated or treated with siRNA against CHC for 48 h at 37 ˚C. **D)** Scan of the same uncropped blot as in panel C) stripped of anti-CHC antibodies and re-labeled with anti-GAPDH antibody as loading control. **E)** Fluorescence micrographs of SV40 (top panel) and GEMs (lower panel) endocytosis in either non-transfected CV1 cells (for SV40) or CV1 cells expressing the 0.036 nM binding affinity GPI-anchored nanobody receptor (for GEMs). Cells were either mock treated (left panels) or treated with CytochalasinD (right panels). CV1 cells were incubated for 1 h with 2 µg of either SV40 or GEMs at 37 ˚C before acid wash and imaging on a spinning disk confocal microscope. Scale bar is 10 µm. **F)** Quantification of the mean intensity fluorescence from the experiment represented in E). Shown are means ± s.e.m from n = 3 independent experiments.

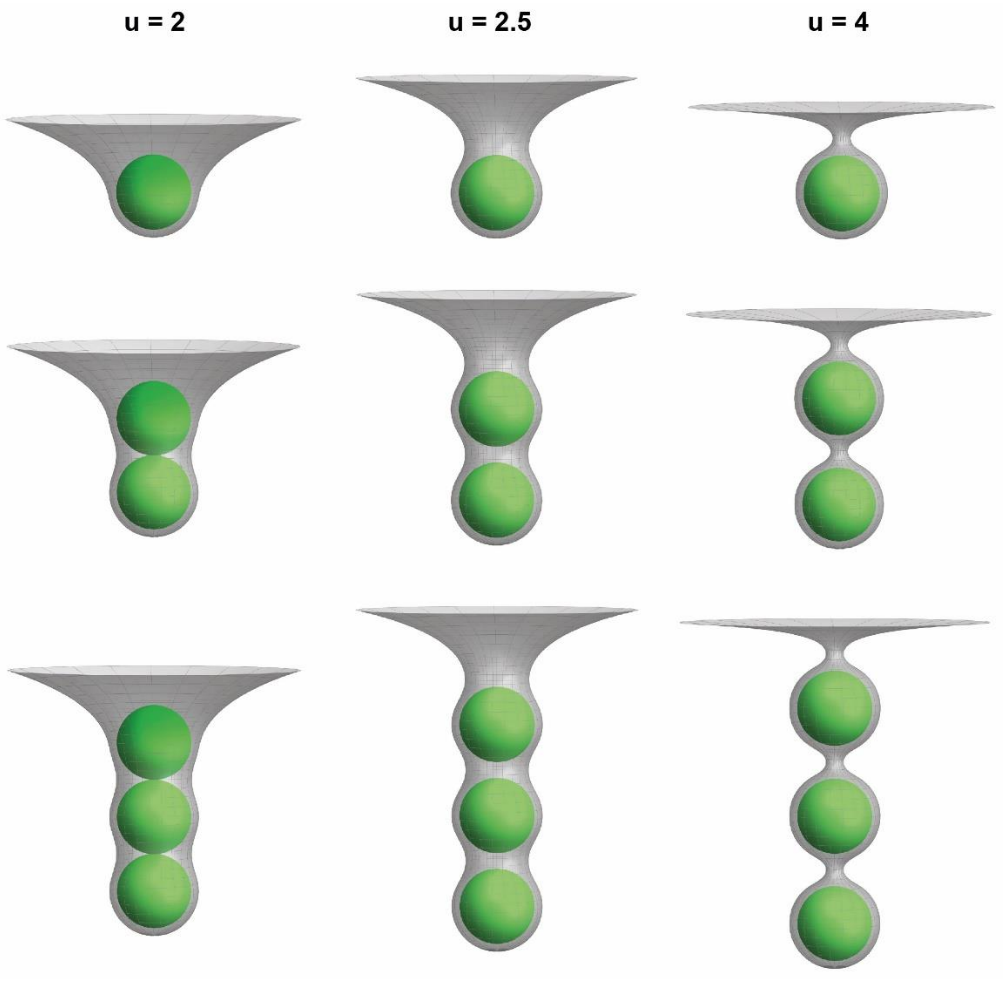

Figure S9. Schematic representation of minimum-energy conformations of GFP GEM particles in dependence on adhesion energy. Minimum-energy conformations for one, two, and three wrapped GEM particles obtained from our model at different values of the rescaled adhesion energy $u = U r^2/\kappa$, where U is the adhesion energy of the particles, r = 23 nm is the membrane midplane radius of a vesicle wrapping a single GEM particle, and κ is the membrane bending rigidity. The value u = 2 corresponds to the wrapping threshold at which a single particle is half wrapped. The GEM particles are represented by green spheres with a radius of 19 nm estimated from the molecular dimensions illustrated in Fig. 5A. The energy gain per particle in Fig. 5C is calculated as the difference between (i) the energy per particle in a long tube with many particles and (ii) the energy for an individually wrapped particle.
