# Supplementary material for: Adhesion energy controls lipid binding-mediated endocytosis
Source: Nat Commun. 2024 Mar 29;15:2767. doi: 10.1038/s41467-024-47109-7 (PMC10980822; doi:10.1038/s41467-024-47109-7)
Supplement: Supplementary file 3 — Description of Additional Supplementary Files [file 41467_2024_47109_MOESM3_ESM.pdf]

Description of Additional Supplementary Files

File name: Supplementary Movie 1

Description: Fluorescence micrographs of GEMs bound to CV1 cells expressing the GPI-anchored anti-GFP nanobody. CV1 cells were incubated for 5 min with 2 µg of GEMs at 37 ˚C before time-course live imaging on a spinning disk confocal microscope. Frames were taken 20 sec apart. Scale bar is 10 µm.

File name: Supplementary Movie 2

Description: Overview of a representative correlative fluorescence and platinum-replica electron microscopy micrograph of plasma membrane sheets generated after unroofing of cells incubated with GEMs (fluorescence signal shown in green).

File name: Supplementary Movie 3

Description: High magnification correlative fluorescence light microscopy and transmission electron microscopy of GEMs internalized in CV-1 cells. CV1 cells were incubated for 1 h with 5 µg of GEMs at 37 ˚C before cryofixation of the sample. GEM positive regions determined from fluorescence microscopy marked in green.
