# Supplementary material for: Adhesion energy controls lipid binding-mediated endocytosis
Source: Nat Commun. 2024 Mar 29;15:2767. doi: 10.1038/s41467-024-47109-7 (PMC10980822; doi:10.1038/s41467-024-47109-7)
Supplement: Supplementary file 7 — Reporting Summary [file 41467_2024_47109_MOESM7_ESM.pdf]

nature portfolio

Corresponding author(s): Helge Ewers
Last updated by author(s): Mar 4, 2024

Reporting Summary

Nature Portfolio wishes to improve the reproducibility of the work that we publish. This form provides structure for consistency and transparency in reporting. For further information on Nature Portfolio policies, see our Editorial Policies and the Editorial Policy Checklist.

Statistics

For all statistical analyses, confirm that the following items are present in the figure legend, table legend, main text, or Methods section.

n/a	Confirmed	
☐	☒	The exact sample size (n) for each experimental group/condition, given as a discrete number and unit of measurement
☐	☒	A statement on whether measurements were taken from distinct samples or whether the same sample was measured repeatedly
☒	☐	The statistical test(s) used AND whether they are one- or two-sided<br>*Only common tests should be described solely by name; describe more complex techniques in the Methods section.*
☒	☐	A description of all covariates tested
☒	☐	A description of any assumptions or corrections, such as tests of normality and adjustment for multiple comparisons
☐	☒	A full description of the statistical parameters including central tendency (e.g. means) or other basic estimates (e.g. regression coefficient) AND variation (e.g. standard deviation) or associated estimates of uncertainty (e.g. confidence intervals)
☒	☐	For null hypothesis testing, the test statistic (e.g. F, t, r) with confidence intervals, effect sizes, degrees of freedom and P value noted<br>*Give P values as exact values whenever suitable.*
☒	☐	For Bayesian analysis, information on the choice of priors and Markov chain Monte Carlo settings
☒	☐	For hierarchical and complex designs, identification of the appropriate level for tests and full reporting of outcomes
☐	☒	Estimates of effect sizes (e.g. Cohen's d, Pearson's r), indicating how they were calculated

Our web collection on statistics for biologists contains articles on many of the points above.

Software and code

Policy information about availability of computer code

Data collection	Spinning disk confocal microscopy images were acquired using MetaMorph version 7.8.6.0 software. Total internal reflection microscopy images were acquired using MicroManager 1.4 Software. Flow cytometry data were acquired using BD FACSDiva version 9.0 Software. Transmission electron microscopy data were acquired using SerialEM version 4.0.0 software. Scanning electron microscopy data was acquired using MAPS version 3.17 software.
Data analysis	Flow cytometry data were analyzed using FlowJo version 10.8.1 Software. Transmission electron tomograms were reconstructed using IMOD version 4.11.12 software. Correlation between fluorescence and transmission electron microscopy data was performed using ec-CLEM plugin in Icy version 2.4.2.0 Software. Scanning electron microscopy data was stitched using Grid/Collection plugin in ImageJ version 1.51. Fluorescence and scanning electron microscopy correlation was performed using BigWarp plugin in ImageJ version 1.51. All microscopy data was analyzed using ImageJ version 1.51. Colocalization analysis was performed in CellProfiler version 4.2.1. All plots were made in OriginPro 2020b. Theoretical modeling was performed in Mathematica13.

For manuscripts utilizing custom algorithms or software that are central to the research but not yet described in published literature, software must be made available to editors and reviewers. We strongly encourage code deposition in a community repository (e.g. GitHub). See the Nature Portfolio guidelines for submitting code & software for further information.

Data

Policy information about availability of data

All manuscripts must include a data availability statement. This statement should provide the following information, where applicable:

- Accession codes, unique identifiers, or web links for publicly available datasets
- A description of any restrictions on data availability
- For clinical datasets or third party data, please ensure that the statement adheres to our policy

The microscopy data shown in the main figures is deposited at https://github.com/AG-Ewers/GEM-project. Raw data supporting the findings of this manuscript are too large to be deposited and are available from the corresponding author upon reasonable request. Source data are provided with this paper.

Research involving human participants, their data, or biological material

Policy information about studies with human participants or human data. See also policy information about sex, gender (identity/presentation), and sexual orientation and race, ethnicity and racism.

Reporting on sex and gender	N/A
Reporting on race, ethnicity, or other socially relevant groupings	N/A
Population characteristics	N/A
Recruitment	N/A
Ethics oversight	N/A

Note that full information on the approval of the study protocol must also be provided in the manuscript.

Field-specific reporting

Please select the one below that is the best fit for your research. If you are not sure, read the appropriate sections before making your selection.

☒ Life sciences ☐ Behavioural & social sciences ☐ Ecological, evolutionary & environmental sciences

For a reference copy of the document with all sections, see nature.com/documents/nr-reporting-summary-flat.pdf

Life sciences study design

All studies must disclose on these points even when the disclosure is negative.

Sample size	The sample size varied depending on the type of experiments and is specified in the figure legends for all experiments contained in this study. No pre study sample size calculations were performed. For all the microscopy experiments, at least 5 cells were imaged per sample, according to the availability of cells and the duration of the experiments. For flow cytometry experiments, at least 5000 cells per sample were imaged. Flow cytometry experiments are highly reliable due to the large number of cells investigated, therefore imaging a few thousands of cells/ sample and repeating the measurement two times was sufficient. For intracellular traffic experiments with CLEM, at least 3 cells were imaged per condition. There was no quantification performed in these experiments. For colocalization experiments with PREM, 3 cells were imaged per sample and repeated twice, as this is a very labor intensive experiment and we provide additional quantitative experiments to support our findings.
Data exclusions	Rounded or dead cells were excluded from microscopy image analysis. Flow cytometry data was gated as described in Figure S4. No data was excluded from electron microscopy experiments.
Replication	All microscopy data were reproduced twice or three times, as indicated in the figure captions. Flow cytometry data were reproduced twice or three times, as indicated in the figure captions. PREM data were reproduced twice.
Randomization	Samples were divided into control groups, where either there was no pharmacological inhibition performed or there was positive/negative effect expected in accordance to previous studies (cited in the main text) and non-control groups, containing the designed artificial system presented in the manuscript.
Blinding	The microscopy colocalization analysis was performed by an automated algorithm in CellProfiler that identified objects in images based on their shape and calculated the overlap between the identified objects in different images. For the flow cytometry experiments, the second independent measurement was performed by the technical assistant of the group that was blinded from the content of the samples.

Behavioural & social sciences study design

All studies must disclose on these points even when the disclosure is negative.

Study description	*Briefly describe the study type including whether data are quantitative, qualitative, or mixed-methods (e.g. qualitative cross-sectional, quantitative experimental, mixed-methods case study).*
Research sample	*State the research sample (e.g. Harvard university undergraduates, villagers in rural India) and provide relevant demographic information (e.g. age, sex) and indicate whether the sample is representative. Provide a rationale for the study sample chosen. For studies involving existing datasets, please describe the dataset and source.*
Sampling strategy	*Describe the sampling procedure (e.g. random, snowball, stratified, convenience). Describe the statistical methods that were used to predetermine sample size OR if no sample-size calculation was performed, describe how sample sizes were chosen and provide a rationale for why these sample sizes are sufficient. For qualitative data, please indicate whether data saturation was considered, and what criteria were used to decide that no further sampling was needed.*
Data collection	*Provide details about the data collection procedure, including the instruments or devices used to record the data (e.g. pen and paper, computer, eye tracker, video or audio equipment) whether anyone was present besides the participant(s) and the researcher, and whether the researcher was blind to experimental condition and/or the study hypothesis during data collection.*
Timing	*Indicate the start and stop dates of data collection. If there is a gap between collection periods, state the dates for each sample cohort.*
Data exclusions	*If no data were excluded from the analyses, state so OR if data were excluded, provide the exact number of exclusions and the rationale behind them, indicating whether exclusion criteria were pre-established.*
Non-participation	*State how many participants dropped out/declined participation and the reason(s) given OR provide response rate OR state that no participants dropped out/declined participation.*
Randomization	*If participants were not allocated into experimental groups, state so OR describe how participants were allocated to groups, and if allocation was not random, describe how covariates were controlled.*

Ecological, evolutionary & environmental sciences study design

All studies must disclose on these points even when the disclosure is negative.

Study description	*Briefly describe the study. For quantitative data include treatment factors and interactions, design structure (e.g. factorial, nested, hierarchical), nature and number of experimental units and replicates.*
Research sample	*Describe the research sample (e.g. a group of tagged Passer domesticus, all Stenocereus thurberi within Organ Pipe Cactus National Monument), and provide a rationale for the sample choice. When relevant, describe the organism taxa, source, sex, age range and any manipulations. State what population the sample is meant to represent when applicable. For studies involving existing datasets, describe the data and its source.*
Sampling strategy	*Note the sampling procedure. Describe the statistical methods that were used to predetermine sample size OR if no sample-size calculation was performed, describe how sample sizes were chosen and provide a rationale for why these sample sizes are sufficient.*
Data collection	*Describe the data collection procedure, including who recorded the data and how.*
Timing and spatial scale	*Indicate the start and stop dates of data collection, noting the frequency and periodicity of sampling and providing a rationale for these choices. If there is a gap between collection periods, state the dates for each sample cohort. Specify the spatial scale from which the data are taken*
Data exclusions	*If no data were excluded from the analyses, state so OR if data were excluded, describe the exclusions and the rationale behind them, indicating whether exclusion criteria were pre-established.*
Reproducibility	*Describe the measures taken to verify the reproducibility of experimental findings. For each experiment, note whether any attempts to repeat the experiment failed OR state that all attempts to repeat the experiment were successful.*
Randomization	*Describe how samples/organisms/participants were allocated into groups. If allocation was not random, describe how covariates were controlled. If this is not relevant to your study, explain why.*
Blinding	*Describe the extent of blinding used during data acquisition and analysis. If blinding was not possible, describe why OR explain why blinding was not relevant to your study.*

Did the study involve field work? ☐ Yes ☐ No

Field work, collection and transport

Field conditions	*Describe the study conditions for field work, providing relevant parameters (e.g. temperature, rainfall).*
Location	*State the location of the sampling or experiment, providing relevant parameters (e.g. latitude and longitude, elevation, water depth).*
Access & import/export	*Describe the efforts you have made to access habitats and to collect and import/export your samples in a responsible manner and in compliance with local, national and international laws, noting any permits that were obtained (give the name of the issuing authority, the date of issue, and any identifying information).*
Disturbance	*Describe any disturbance caused by the study and how it was minimized.*

Reporting for specific materials, systems and methods

We require information from authors about some types of materials, experimental systems and methods used in many studies. Here, indicate whether each material, system or method listed is relevant to your study. If you are not sure if a list item applies to your research, read the appropriate section before selecting a response.

Materials & experimental systems

n/a	Involved in the study	
☐	☒	Antibodies
☐	☒	Eukaryotic cell lines
☒	☐	Palaeontology and archaeology
☒	☐	Animals and other organisms
☒	☐	Clinical data
☒	☐	Dual use research of concern
☒	☐	Plants

Methods

n/a	Involved in the study	
☒	☐	ChIP-seq
☐	☒	Flow cytometry
☒	☐	MRI-based neuroimaging

Antibodies

Antibodies used	Clathrin Heavy Chain (P1663) Antibody #2410 Cell Signaling Technologies; Goat anti-rabbit IgG Alexa Fluor 568 #A-11011 Invitrogen; Goat anti-rabbit HRP antibody 31462 Invitrogen; Anti-GAPDH antibody [6C5] - Loading Control ab8245 Abcam
Validation	Clathrin Heavy Chain antibody was used in a Western Blot in the manuscript to determine the amount of Clathrin Heavy Chain expression in cells. On the manufacturer's website, confocal immunofluorescent analysis of HeLa cells labeled with Clathrin Heavy Chain antibody shows a typical clathrin pattern and a Western blot analysis of extracts from SHSY5Y, Neuro2A, PC12 and COS cells, using Clathrin Heavy Chain antibody displays a band at the correct molecular weight.<br><br>Goat anti-rabbit IgG Alexa Fluor 568 was validated by Invitrogen by multiple immunostainings, revealing a good colocalization with the primary antibodies against the specific organelles used in the assays.<br><br>Goat anti-rabbit HRP antibody was validated by Invitrogen on a Western Blot analysis revealing the correct cross-absorption with the primary antibody used in each assay at the appropriate molecular weight.<br><br>Anti-GAPDH antibody [6C5] - Loading Control was validated by Abcam on a Western blot analysis revealing a band at the correct molecular weight. In addition, immunofluorescence analysis performed by the manufacturer reveals a cytosolic localization of the antibody.<br><br>All the manufacturers report citations on their websites for all the antibodies used in this study.

Eukaryotic cell lines

Policy information about cell lines and Sex and Gender in Research

Cell line source(s)	CV1 cells were purchased from ATCC (CCL-70). NRK49F cells were purchased from ATCC (CRL-1570).
Authentication	Cell lines were not authentificated.
Mycoplasma contamination	Cells were regularly tested for mycoplasma contamination using a PCR-based test and were found negative each time.
Commonly misidentified lines (See ICLAC register)	No commonly misidentified cell lines were used in this study.

Flow Cytometry

Plots

Confirm that:

☒ The axis labels state the marker and fluorochrome used (e.g. CD4-FITC).

☒ The axis scales are clearly visible. Include numbers along axes only for bottom left plot of group (a 'group' is an analysis of identical markers).

☐ All plots are contour plots with outliers or pseudocolor plots.

☒ A numerical value for number of cells or percentage (with statistics) is provided.

Methodology

Sample preparation	Cells were either detached using Trypsin or Accutase, as described in the Methods section. Cells were spun and supernatant removed. Cells were resuspended in cellular medium prior to measurement.
Instrument	BD FACSCanto Flow Cytometry System
Software	BD FACSDiva Software
Cell population abundance	The final cell populations after gating ranged between 0.1%-93%.
Gating strategy	In brief, live cell population was always selected first from SSC-A vs. FSC-A plots. From the selected live cell population, doublets and aggregates were always removed from the FSC-H vs. FSC-A plots. Next, the RFP positive population gate was selected according to the RFP negative control from the SSC-A vs. 561 nm plots. Next, the GFP positive population gate was selected according to the RFP positive, GFP negative control from the SSC-A vs. 488 nm plots. The gating strategy is described in more detail in Figure S4.

☒ Tick this box to confirm that a figure exemplifying the gating strategy is provided in the Supplementary Information.
